# Mesenchymal stem cell–derived small extracellular vesicles (sEVs) as a therapy for sepsis-related liver injury: evidence from a systematic review and meta-analysis

**DOI:** 10.3389/fphar.2025.1707784

**Published:** 2025-11-27

**Authors:** Yan Pan, Youting Xu, Ting Shui, Jiao Hong, Xuan Lu, Huilin Chen

**Affiliations:** Department of Blood Transfusion, The Quzhou Affiliated Hospital of Wenzhou Medical University, Quzhou People’s Hospital, Quzhou, Zhejiang, China

**Keywords:** mesenchymal stem cell-derived small extracellular vesicles, sepsis, liver injury, animal models, meta-analysis

## Abstract

**Background:**

Sepsis-induced liver injury (SILI) is critical in the progression of high morbidity and mortality associated with sepsis which ends in hepatic dysfunction and multi-organ failure. Mesenchymal stem cell–derived small extracellular vesicles (MSC-sEVs) are valued for their anti-inflammatory and regenerative potential as favorable strategy. The present systematic review and meta-analysis aimed to assess the effect of MSC- sEVs in rodent models with SILI.

**Methods:**

A comprehensive systematic search was carried out in the PubMed, Web of Science, Embase, Scopus, and the Cochrane Library through April 2025. All published studies in relation to the effect of MSC- sEVs in rodent models were included. Pooled standardized mean differences (SMDs) or odds ratios (ORs) with 95% confidence intervals (CIs) were calculated for study outcomes.

**Results:**

Ten studies were included in the present study. MSC- sEVs significantly reduced ALT (SMD = −2.49, 95% CI: 3.37, −1.62), AST (SMD = −1.97, 95% CI: 3.32, −0.62), reduced pro-inflammatory cytokines (TNF-α: SMD = −5.23, 95% CI: 7.05, −3.41; IL-6: SMD = −5.00, 95% CI: 7.36, −2.64), and increased survival (OR = 6.11, 95% CI: 2.20–16.98; P = 0.001). No significant effects were observed for IL-10 (SMD = −3.39, 95% CI: 9.47, 2.69) or NLR (SMD = −0.65, 95% CI: 1.75, 0.45). Subgroup analyses illustrated that overall efficacy of treatment may vary dependent to source of sEVs, route of administration, and induction methods.

**Conclusion:**

MSC- sEVs is able to improve liver function, inflammation, and survival rate in rodent sepsis model. These findings suggest that MSC- sEVs could be considered as therapeutic strategy for sepsis. These findings not only quantify the effect size of MSC- sEVs but also provide methodological insights for preclinical studies and guide future translational research.

## 1 Introduction

Sepsis is characterized with host immune responses to infection contributing to systemic inflammation (Mehdi et al., 2025; Jacob et al., 2025). Its high morbidity and mortality worldwide pose a major challenge to global public health (Prest et al., 2022; Bauer et al., 2020). The liver serves as a key organ in the pathogenesis of sepsis (Yan et al., 2014; Beyer et al., 2022). Sepsis-associated liver injury (SILI) has been associated with liver dysfunction and increased risk of multiple organ dysfunction syndrome (MODS) (Strnad et al., 2017; Kobashi et al., 2013; Chen et al., 2024; Guo et al., 2025).

Evidence indicates that mesenchymal stem cells (MSCs) could be considered as a research hotspot in regenerative medicine. Several beneficial properties have been attributed to the MSCs such as immunomodulatory properties, regenerative functions (Via et al., 2012; Sart et al., 2014; Naji et al., 2019; Marino et al., 2019). In this context, small extracellular vesicles (sEVs) have been known as key mediators which transfer transferring bioactive molecules (Park et al., 2019; Hashemian et al., 2020). Likewise, Yue et al. and Feng et al. deomstrated that MSC-derived sEVs are potent enough to improve renal ischemia-reperfusion injury (Yue et al., 2022; Feng et al., 2024). Similarly, improve lung injury was shown by MSC-derived sEVs (Cui et al., 2024; Li et al., 2019). This favorable features are possible through several mechanisms such as inhibition of NF-κB signaling pathway (Sun et al., 2021), regulation of macrophage polarization (Liu et al., 2020), and promoted proliferation (Tan et al., 2014).

Taken together, these findings suggest that a significant gap remains in understanding the protective effects of MSC-derived sEVs in sepsis-induced liver injury models. In this regard, discrepancies in animal models, cell sources, sEV isolation methods, and dosage have led to inconsistent findings across studies. However, this systematic review and meta-analysis have been conducted based on the SYRCLE risk-of-bias tool to illustrate the efficacy of MSC-derived sEVs in treating SILI.

## 2 Methods

### 2.1 Ethic statement

The study protocol has been registered (CRD420251184561) in the International Prospective Register of Systematic Reviews (PROSPERO) database. Also, this study is conducted based on Preferred Reporting Items for Systematic Reviews and Meta-Analyses (PRISMA) guidelines and adheres to the Meta-analysis of Observational Studies in Epidemiology (MOOSE).

### 2.2 Eligibility criteria

All inclusion criteria of the study is performed based on the PICOS as follow: (P: population) rodent models of sepsis; (I: intervention) sEVs from various types of mesenchymal stem cells (C: comparison) studies with comparison groups, (O: outcomes) such as: liver function tests (ALT, AST), inflammatory markers (TNF-α, IL-6, IL-10), survival, and the neutrophil-to-lymphocyte ratio (NLR) and (S: study design) randomized control study and MSC-sEVs treated group vs. sepsis control group.

### 2.3 Search strategy

**FIGURE 1:**
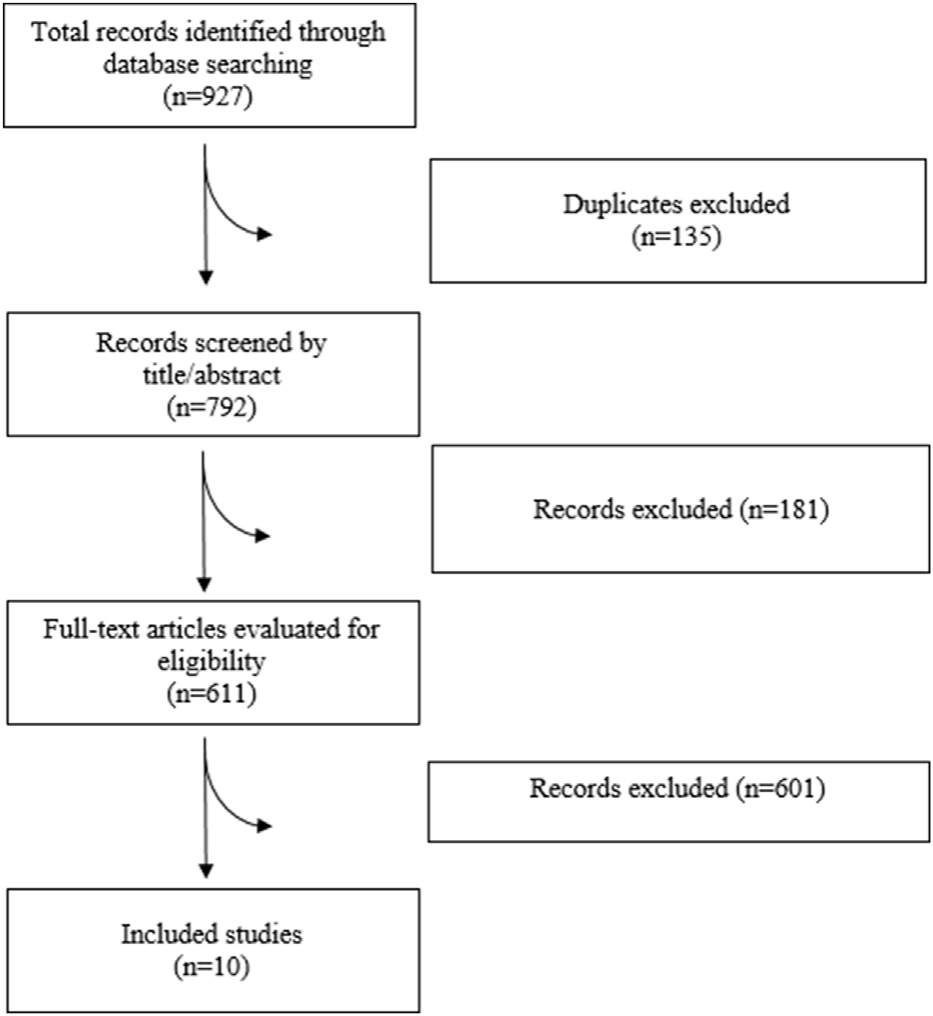
Flowchart of study selection process.

A comprehensive systematic search was performed in PubMed, Web of Science, Embase, Scopus, and Cochrane Library to identify studies that met our predefined criteria up to April 2025. There was no limitation in the publication date or language. Search strategy and study selection process have been provided in Supplementary Table S1 and Figure 1, respectively.

### 2.4 Data extraction

Screening process and data extraction have been done by two investigators independently. Any discrepancies were resolved through discussion, and when consensus could not be achieved, a third researcher was consulted to make the final decision. Basic characteristics of included studies such as study design, sample size, animal model, exosome characteristics, administration route and treatment duration, and mean and SD for included outcomes.

### 2.5 Statistical analysis

The data analysis of this study was conducted using RevMan 5.4 and STATA 18.0 software. Subgroup analyses were based on the MSC-Exos source (human umbilical cord mesenchymal stem cells (hUC-MSCs), human umbilical cord Wharton’s gel mesenchymal stem cells (WJ-MSCs), others (MSC-derived sEVs obtained from alternative tissue sources, such as bone marrow, adipose tissue)), administration route (tail vein injection and intraperitoneal injection), and induction method (CLP, LPS). All outcomes are presented with pooled SMD and 95% confidence interval (CI). The degree of heterogeneity was evaluated using the I^2^ statistic. Publication bias was evaluated using the Egger regression model and visualized using a funnel plot.

### 2.6 Risk of bias and quality assessment

Two independent reviewers evaluated the risk of bias using Systematic Review Centre for Laboratory Animal Experimentation (SYRCLE) tool and the National Institutes of Health (NIH) guidelines.

## 3 Result

### 3.1 Study characteristics

A total of 927 articles were identified through the literature search, of which 10 met the inclusion criteria1 (Chang et al., 2021; Eshghi et al., 2022; Cai et al., 2023; Rahnama et al., 2023; Ahangari et al., 2024; Pei et al., 2024; Shahi et al., 2024; Yuan et al., 2023; Chang et al., 2018, Song et al., 2017). Basic characteristics of included studies are provided in the Table 1.

**TABLE 1:** Characteristics of the included studies.

First author (Year)	Animal	Number	Duration	Source of sEVs	Induction method	Administration route	MCS-sEVs charateristics (surface marker)
Shahi et al. (2024)	C57BL/6 mice	Sham group (8), CLP group (8), CLP + Young MSC-exo group (8), CLP + Old MSC-exo group (8)	3 Days	Adipose tissue-derived stromal cells (ADSC)	CLP	Intraperitoneal injection	CD105、CD29、CD90
Ahangari et al. (2024)	C57BL/6 mice	Sham group (5), CLP group (5), exosome group (5)	1 Day	WJ-MSCs	CLP	Caudal vein	CD9, CD63, CD81
Pei et al. (2024)	C57BL/6 mice	Control group (4), LPS group (4), MSC-exo group (4)	1 Day	hUC-MSCs	LPS	Caudal vein	CD9, CD63, CD81
Cai et al. (2023)	C57BL/6 mice	CLP group (10), CLP + exosome group (10)	7 Days	hUC-MSCs	CLP	Caudal vein	CD9、CD63、TSG101
Rahnama et al. (2023)	C57BL/6 mice	Normal group (8), Sham group (8), PBS/Ecoli: Group (8), exosome group (8)	7 Days	USSCs	PBS/Ecoli	Caudal vein	CD9、CD63
Yuan et al. (2023)	C57BL/6 mice	Control group (6), CLP group (6), exosome group (6)	-	BMMSCs	CLP	Caudal vein	CD63、CD90、HSP70
Eshghi et al. (2022)	BALB/C mice	Normal group (5), LPS + PBS group (12), LPS + exosome group (16)	2 Days	WJ-MSCs	LPS	Caudal vein	CD63、CD81
Chang et al. (2021)	C57BL/6 mice	HFD group (9), HFDLPS group (12), exosome treatment group (9)	2 Days	pcMSCs	LPS	Intraperitoneal injection	CD63、CD9
Chang et al. (2018)	SD rats	Sham control group (16), sepsis syndrome group (19), Healthy ADMSC-derived exosomes group (18), Apoptotic ADMSC-derived exosomes group (18)	5 Days	Adipose tissue-derived stromal cells (ADSC)	CLP	Caudal vein	CD63、TSG101
Song et al. (2017)	C57BL/6 mice	LPS group (10), MSC-exo group (12), βMSC-exo group (13)	7 Days	hUC-MSCs	CLP	Caudal vein	CD63、Alix

Abbreviations: CLP, cecal ligation and puncture; LPS, lipopolysaccharide; MSC-exo, Mesenchymal stem cells-derived exosome; ALT, alanine aminotransferase; AST, aspartate aminotransferase; TNF-α, Tumor necrosis factor-α; IL-6, Interleukin 6; IL-10, Interleukin 10; NLR, Neutrophils-to-lymphocytes ratio; hUC-MSCs, Human umbilical Cord; ADMSC, Adipose-derived mesenchymal stem cell; TSG101, Anti-tumor susceptibility gene-101; HFD, high fat diet; pcMSCs, Human placenta choriodecidual membrane-derived MSCs; WJ-MSCs, Wharton’s jelly-derived MSCs; USSC, unrestricted somatic stem cells; BMMSCs. bone marrow mesenchymal stem cells.

### 3.2 Bias and quality assessment

Table 2 presents the bias risk assessment for each study across various domains. The following domains including: sequence generation, baseline characteristics, allocation concealment, randomization of interventions, and blinding of outcome assessment were scored as lower risk of bias. However, incomplete outcome data, selective reporting, and other potential sources of bias domains were rated as unclear risk of bias.

**TABLE 2:** Risk assessment of bias in mesenchymal stem cell exosome therapy in animal models of sepsis.

First author (year)	Selection bias (Sequence generation)	Selection bias (Baseline characteristics)	Selection bias (Allocation concealment)	Performance bias (Random housing)	Performance bias (Blinding of interventions)	Detection bias (Random outcome assessment)	Detection bias (Blinding of outcome assessment)	Attrition bias	Reporting bias	Other
Shahi et al. (2024)	U	L	U	L	L	U	L	U	L	L
Ahangari et al. (2024)	U	L	U	L	L	U	L	L	L	
Pei et al. (2024)	L	L	U	L	L	L	U	L	L	L
Cai et al. (2023)	U	U	U	L	L	U	L	L	L	L
Rahnama et al. (2023)	U	L	U	L	L	U	L	L	L	L
Eshghi et al. (2022)	U	L	U	L	U	U	L	L	L	H
Chang et al. (2021)	U	H	U	L	U	U	L	U	L	L
Chang et al. (2018)	U	L	U	L	L	U	L	L	L	L
Yuan et al. (2023)	L	L	U	L	L	U	L	L	L	L
Song et al. (2017)	U	L	U	L	U	U	L	L	L	L

### 3.3 Effects of MSC-Exos on septic related injury model

**FIGURE 2:**
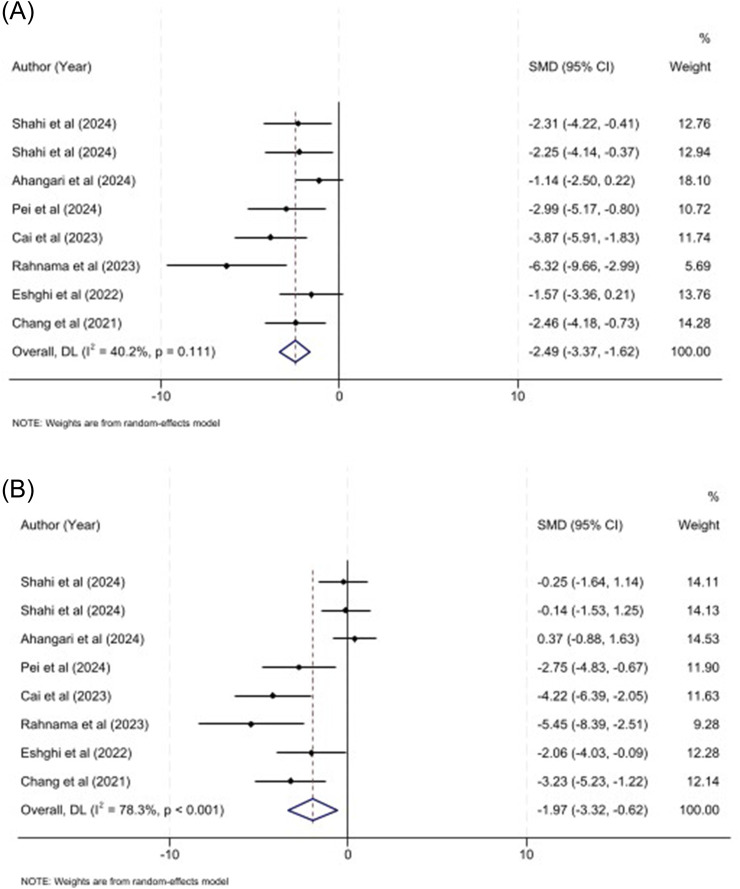
**(A)** Forest plot for the impact of MSC-sEVs on ALT in sepsis models. **(B)** Forest plot for the impact of MSC-sEVs on AST in sepsis models.

ALT and AST biomarkers have been known as a key indicator in the sepsis-induced hepatocellular injuries. Pooled effect of eight studies involving 73 mice demonstrated that MSC-sEVs significantly decreased ALT level (SMD: −2.49, 95% CI: −3.37, −1.62, P < 0.001; I^2^ = 40.2%; P = 0.111) (Figure 2A) and AST (SMD: −1.97, 95% CI: −3.32, −0.62, P = 0.004; I^2^ = 78.3%; P < 0.001) level (Figure 2B). These findings indicate that MSC-sEVs treatment may alleviate hepatocellular damage in rodent model. Subgroup analyses demonstrated both sources of MSC-sEVs (hUC-MSCs, or WJ-MSCs), both administration route (tail vein injection and intraperitoneal injection), and induction method (CLP, LPS) are able to decrease ALT level in mice model (P < 0.05). While, just hUC, LPS, and caudal vein were accompanied with significant decrease in the AST level (Table 3).

**TABLE 3:** Subgroup analyses for the comparison between outcomes.

Outcomes	NO	ES (95% CI)	P-within	*I* ^2^ (%)	P-heterogeneity
ALT					
Source of sEVs					
hUC	2	−3.46 (−4.95, −1.97)	<0.001	0.0	0.564
WJ	2	−1.30 (−2.38, −0.22)	0.019	0.703	0.703
Others	4	−2.86 (−4.20, −1.52)	<0.001	39.8	0.173
Administration route					
Intraperitoneal	4	−2.86 (−4.20, −1.52)	<0.001	39.8	0.173
Caudal vein	4	−2.22 (−3.47, −0.97)	0.001	47.9	0.124
Induction method					
CLP	4	−2.23 (−3.36, −1.10)	<0.001	38.6	0.180
LPS	4	−2.90 (−4.44, −1.36)	<0.001	51.5	0.103
AST					
Source of sEVs					
hUC	2	−3.46 (−4.96, −1.95)	<0.001	0.0	0.339
WJ	2	−0.72 (−3.09, 1.65)	0.553	76.1	0.041
Others	4	−1.96 (−4.04, 0.11)	0.063	81.1	0.001
Administration route					
Intraperitoneal	3	−1.06 (−2.76, 0.65)	0.224	71.7	0.029
Caudal vein	5	−2.64 (−4.76, −0.52)	0.015	82.6	<0.001
Induction method					
CLP	4	−0.86 (−2.45, 0.73)	0.289	77.8	0.004
LPS	4	−3.08 (−4.29, −1.88)	<0.001	17.4	0.304
TNF-α					
Source of sEVs					
hUC	3	−8.91 (−16.10, −1.72)	0.015	90.9	<0.001
WJ	2	−6.84 (−17.61, 3.94)	0.214	84.9	0.010
Others	6	−4.55 (−6.51, −2.59)	<0.001	69.1	0.006
Administration route					
Intraperitoneal	3	−3.71 (−6.08, −1.34)	0.002	59.6	0.084
Caudal vein	8	−6.30 (−8.87, −3.74)	<0.001	83.1	<0.001
Induction method					
CLP	7	−5.31 (−7.49, −3.13)	<0.001	77.1	<0.001
LPS	4	−5.71 (−9.59, −1.83)	0.004	83.1	<0.001
IL-6					
Source of sEVs					
hUC	1	−6.82 (−10.92, −2.72)	0.001	0.0	<0.001
WJ	1	−8.27 (−13.60, −2.93)	0.002	0.0	<0.001
Others	3	−4.01 (−6.59, −1.43)	0.002	73.0	0.024
Administration route					
Intraperitoneal	1	−1.95 (−3.52, −0.39)	0.014	0.0	<0.001
Caudal vein	4	−5.84 (−7.50, −4.18)	<0.001	0.0	0.618
Induction method					
CLP	1	−6.06 (−8.94, −3.17)	<0.001	0.0	<0.001
LPS	4	−4.76 (−7.54, −1.99)	0.001	70.2	0.018
IL-10					
Source of sEVs					
hUC	3	3.69 (−4.32, 11.71)	0.366	92.6	<0.001
Others	3	−8.31 (−14.93, −1.69)	0.014	83.4	0.002
Administration route					
Intraperitoneal	2	−4.60 (−6.71, −2.49)	<0.001	0.0	0.461
Caudal vein	4	−3.09 (−12.37, 6.18)	0.514	93.5	<0.001
Induction method					
CLP	4	−1.43 (−10.31, 7.46)	0.753	94.9	<0.001
LPS	2	−14.20 (−47.79, 19.40)	0.408	94.4	<0.001
NLR					
Source of sEVs					
WJ	2	0.13 (−1.03, 1.29)	0.831	26.3	0.244
Others	2	−1.59 (−2.77, −0.42)	0.008	0.0	0.627
Administration route					
Intraperitoneal	2	−1.59 (−2.77, −0.42)	0.008	0.0	0.627
Caudal vein	2	0.13 (−1.03, 1.29)	0.831	26.3	0.244
Induction method					
CLP	3	−1.04 (−1.94, −0.14)	0.024	8.2	0.336
LPS	1	0.83 (−0.75, 2.41)	0.305	0.0	<0.001
Survival rate					
Source of sEVs					
hUC	3	4.84 (0.24, 96.98)		74.5	0.020
Others	7	6.14 (2.34, 16.15)		20.4	0.274
Administration route					
Intraperitoneal	3	23.94 (3.94, 145.57)		0.0	0.901
Caudal vein	7	4.11 (1.29, 13.08)		49.1	0.067
Induction method					
CLP	8	4.59 (1.54, 13.65)		45.7	0.075
LPS	2	28.36 (3.12, 257.75)		0.0	0.708

**FIGURE 3:**
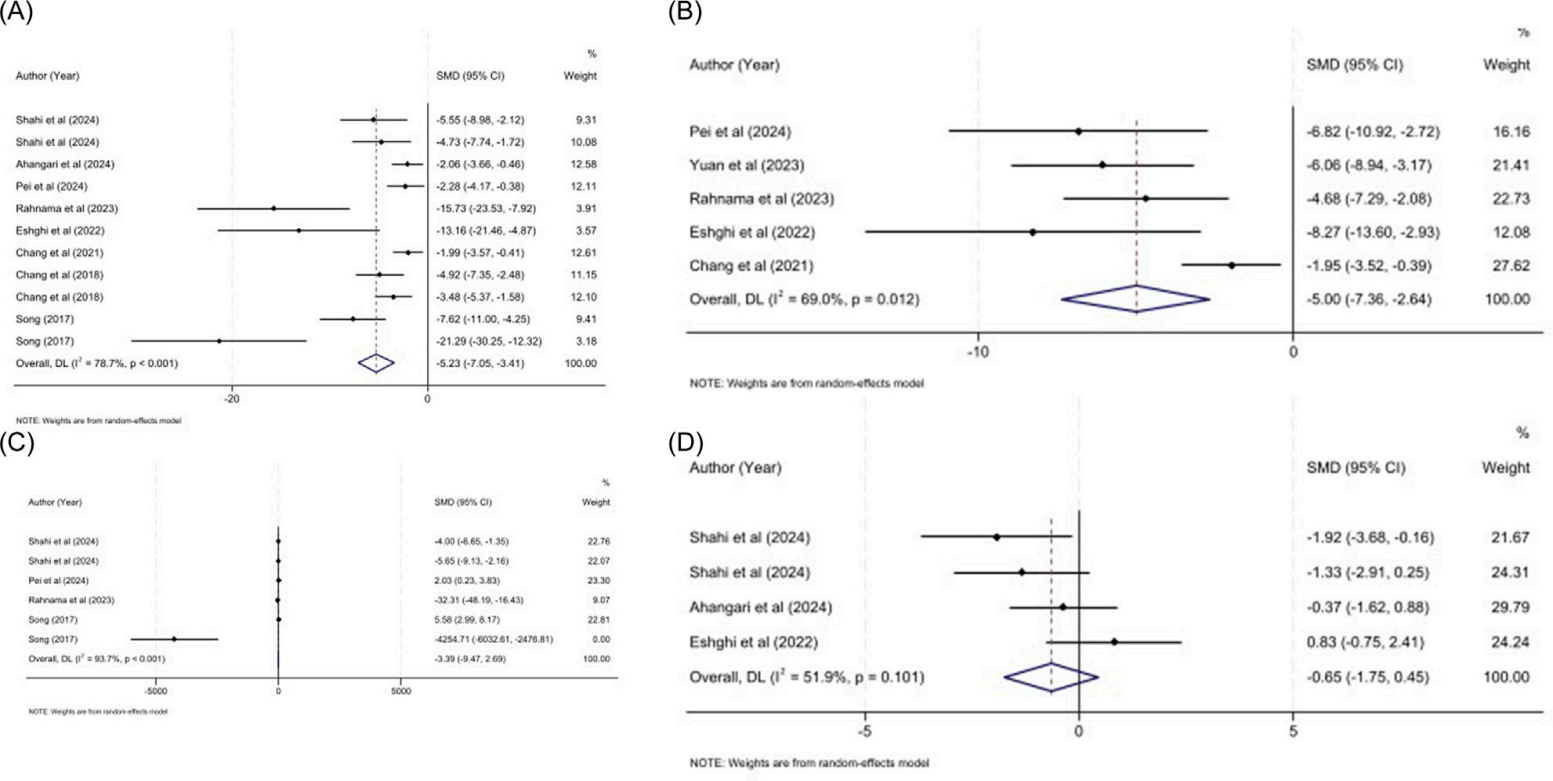
**(A)** Forest plot for the impact of MSC-Exos on TNF-α in sepsis models. **(B)** Forest plot for the impact of MSC-Exos on IL-6 in sepsis models. **(C)** Forest plot for the impact of MSC-Exos on IL-10 in sepsis models. **(D)** Forest plot for the impact of MSC-Exos on NLR in sepsis models.

Moreover, sepsis is characterized by elevated inflammatory responses. Likewise, TNF-α and IL-6 are pro-inflammatory cytokines are involved in the systemic inflammation in SILIs. Combined effect of eleven studies (111 animal) investigating the effect of MSC-sEVs on TNF-α showed that MSC-sEVs are potent to reduce the TNF-α level compared to sepsis induced animals with no interventions (SMD: −5.23, 95% CI: −7.05, −3.41, P < 0.001; I^2^ = 78.7%; P < 0.001) (Figure 3A). Subgroup analysis was performed based on the source of sEVs, administration route and induction method for inflammatory markers. Likewise, all subgroups of both administration route (tail vein injection and intraperitoneal injection), and induction method (CLP, LPS) showed significant reduction in the TNF-α level. A significant reduction was also observed in the hUC subgroup (Table 3). Similarly, it had beneficial effect on the IL-6 in the pooled five studies (SMD: −5.00, 95% CI: −7.36, −2.64, P < 0.001; I^2^ = 69.0%; P = 0.012) (Figure 3B.). Subgroup analysis revealed that all subgroups, regardless of the sEV source, administration route, or induction method, were associated with a significant decrease in IL-6 levels (Table 3). While, MSC-sEVs failed to exert favorable effects on the IL-10 (SMD: −3.39, 95% CI: −9.47, 2.69, P = 0.274; I^2^ = 93.7%; P < 0.001) (Figure 3C.) and NLR (SMD: −0.65, 95% CI: −1.75, 0.45, P = 0.248; I^2^ = 51.9%; P = 0.110) value significantly (Figure 3D).

**FIGURE 4:**
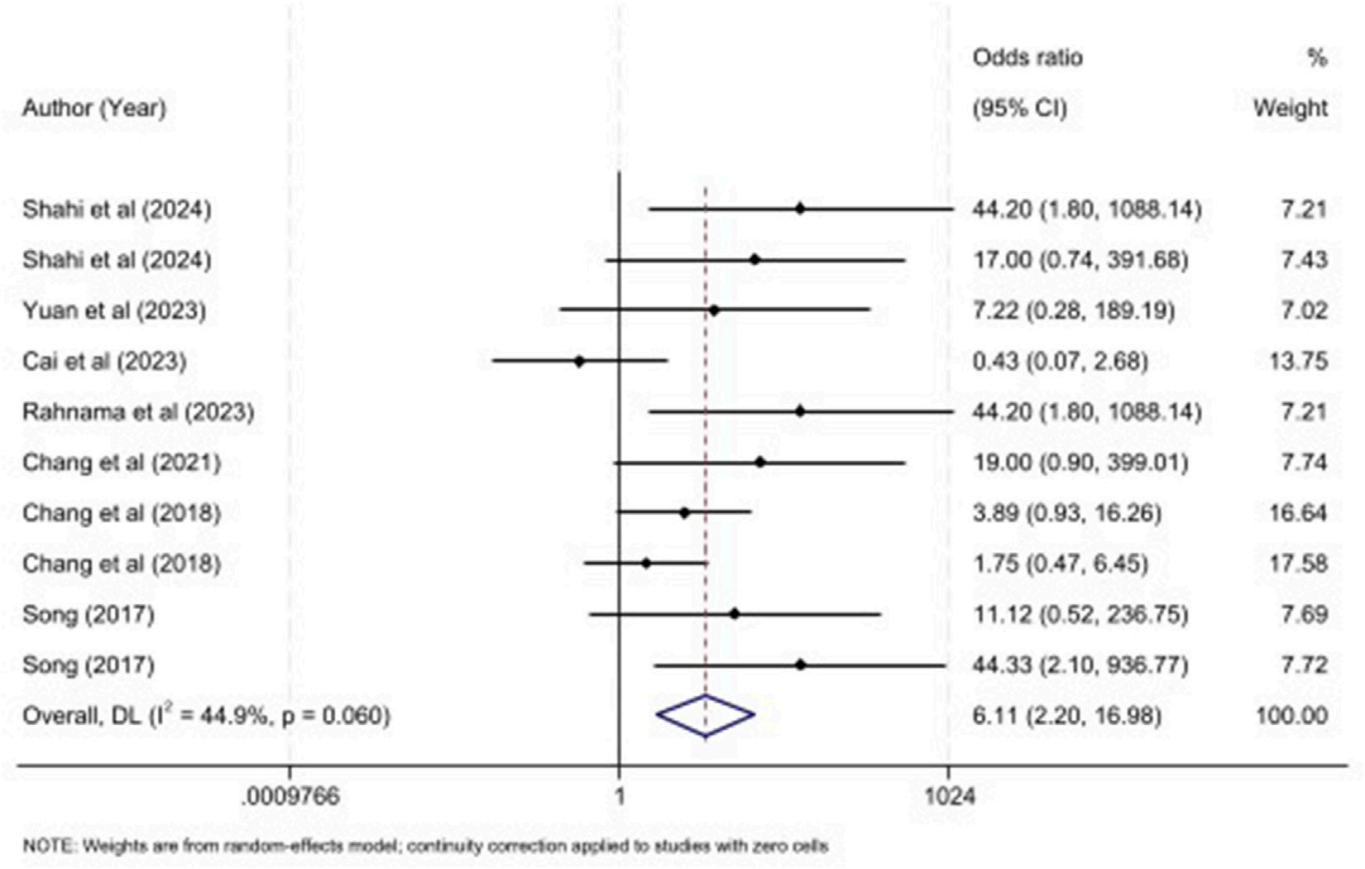
Forest plot for the impact of MSC-Exos on survival rate in sepsis models.

Furthermore, survival rate serves as a comprehensive indicator of treatment efficacy in SILIs, as, it is accompanied with improved diseases condition including functional efficacy and inflammatory state. Elevated survival rate could be translated to multiorgan failure and hepatocellular dysfunction which has been triggered by sepsis ten studies encompassing 220 animals with sepsis and treated with MSC- sEVs illustrated that MSC- sEVs could increase survival rate significantly (OR: 6.11, 95% CI: 2.20, 16.98, P = 0.001; I^2^ = 44.9%; P = 0.060) (Figure 4). In this regard, subgroup analysis revealed that, hUC was no associated with increased OR of survival rate in the rodent model, while alternative tissue sources, such as bone marrow, adipose tissue were associated with elevated OR (Table 3). Also, both intraperitoneal and caudal vein route and both induction methods such as CLP or LPS showed increased OR in pooled studies (Table 3).

### 3.4 Sensitivity analysis and publication bias

The sensitivity analyses demonstrated that leave-one-out approach did not affect the overall pooled results for all study outcomes including ALT, AST, TNF-α, IL-6, IL-10, and survival rate. Exclusion of Eshghi et al.’s study resulted in a significant change in the pooled effect on NLR (Supplementary Figures S2–S7).

Publication bias using Egger’s tests along with funnel plots was assessed. There was evidence of publication bias regarding ALT (egger’s test = 0.001), AST (egger’s test = <0.001), TNF-α (egger’s test = <0.001), survival rate (egger’s test = 0.012). However, the trim-and-fill analysis indicated no significant adjustments. In contrast, for IL-10 (egger’s test = 0.060), and NLR (egger’s test = 0.625) the funnel plots were symmetrical (Supplementary Figures S8–S11).

## 4 Discussion

The present systematic review and meta-analysis provide a comprehensive effect of MSC-sEVs in rodent models of sepsis-induced liver injury. The findings demonstrated that MSC- sEVs treatment is able to promote liver function (ALT, AST), alleviate the systemic inflammation (TNF-α, IL-6) and increase survival rates in rodent model.

It has been revealed that MSC-sEVs treatment is potent enough to improved liver function, as evidenced by the reduction in ALT and AST levels. Promoted sepsis-induced inflammation in hepatocellulars represent liver injury. Likewise, it seems that MSC-sEV administration may have protective effects against the liver function. Evidence suggests that protective effects of may be triggered by suppressed NF-κB and TLR4 signaling pathways (Peng et al., 2023).

Moreover, it is able to alleviate inflammatory state by decreasing TNF-α and IL-6, and In addition, subgroup analyses provided insightful effects of MSC-sEVs across source of sEVs, adminstration route and induction methods. It sems that MSC- sEVs may have therapeutic effects to modulate inflammation and promote tissue repair. These anti-inflammatory properties may be mediated through the following mechanisms: the modulation of inflammatory signaling pathways (suppression of NF-κB and TLR4 signaling) and attenuation of liver injury (Jin et al., 2025), the promotion of tissue repair via bioactive molecules that enhance hepatocyte proliferation and differentiation (Aguiar Koga et al., 2023), and the activation of protective intracellular signaling cascades that support cell survival and functional recovery (Li et al., 2024).

A moderate to high degree of heterogeneity was observed among the included studies for all study outcomes. Accordingly, subgroup analysis was performed to investigate the impact of factors such as modeling methods, administration routes, and exosome sources on therapeutic efficacy. The results indicated that various source of sEVs (hUC or WJ or other), different modeling methods (CLP or. LPS) and administration routes (intraperitoneal injection or tail vein injection) had distinct effects on specific indicators.

ALT was decreased irrespective of MSC-sEVs source, injection route, or induction method. While, significant improvement in AST was observed in the hUC, caudal vein, and LPS method. Additionally, hUC MSC-derived sEVs (hUC-MSCs) appeared more effective than other sources with both injection route in modulation of inflammatory markers.

Although this study provides a comprehensive evaluation of MSC-sEVs therapy in sepsis-induced liver injury models, several limitations remain. First, smaller sample size affects statistical power and limits the generalizability of the findings. Second, there is a major gap in relation to long-term studies. Third, it is better to interpret the results with caution regarding publication bias in term of ALT, AST, TNF-α, I_6, and survival rate. Taken together, these findings warrant further studies with larger sample sizes and investigations of dose–response relationships.

## 5 Conclusion

This study highlights the significant therapeutic potential of MSCs-derived sEVs in the sepsis-induced liver injury in rodent model. Moreover, it has been shown that MSCs-derived sEVs is able to improve liver function, attenuate inflammatory responses, and enhance survival rates. Furthermore, attention should be given to the source of sEVs, administration routes, and induction methods, as these factors may influence therapeutic efficacy.

## Data Availability

The datasets presented in this study can be found in online repositories. The names of the repository/repositories and accession number(s) can be found in the article/Supplementary Material.
