# Supplementary material for: Mesenchymal stem cell–derived small extracellular vesicles (sEVs) as a therapy for sepsis-related liver injury: evidence from a systematic review and meta-analysis
Source: Front Pharmacol. 2025 Nov 27;16:1707784. doi: 10.3389/fphar.2025.1707784 (PMC12695610; doi:10.3389/fphar.2025.1707784)
Supplement: Supplementary file 1 [file DataSheet1.pdf]

Supplementary material

Mesenchymal Stem Cell–Derived Small Extracellular Vesicles (sEVs) as a Therapy for Sepsis-Related Liver Injury: Evidence from a Systematic Review and Meta-Analysis

Supplementary Table 1. Search strategy in databases**.**

PubMed

No	Query
10	#8 AND #1 AND (#4 OR #6 OR #7) AND (#2 OR #3) AND #5
9	#1 AND (#4 OR #6 OR #7) AND (#2 OR #3) AND #5
8	(controlled clinical trial[pt] OR randomized controlled trial[pt] OR randomized[tiab] OR drug therapy[tiab] OR trial[tiab] OR drug therapy[sh] OR randomly[tiab] OR groups[tiab]) OR (RCT[tiab]) OR (randomized trial[tiab]) OR (random allocation[tiab]) OR (random assignment[tiab]) OR (placebo -controlled trial[tiab]) OR (double -blind trial[tiab]) OR (single - blind trial[tiab]) OR (open -label trial[tiab]) OR (parallel -group trial[tiab]) OR (crossover trial[tiab]) OR (pharmacotherapy[tiab]) OR (medication[tiab]) OR (treatment[tiab]) OR (intervention[tiab]) OR (therapeutic trial[tiab]) OR (clinical study[tiab]) OR (research trial[tiab]) OR (treatment group[tiab]) OR (control group[tiab]) OR (prospective study[tiab]) OR (retrospective study[tiab]) OR (observational study[tiab]) OR (cohort study[tiab]) OR (case - control study[tiab]) OR (cross -sectional study[tiab]) OR (longitudinal study[tiab]) OR (interventional study[tiab]) OR (non -randomized trial[tiab]) OR (quasi -experimental trial[tiab]) OR (pilot trial[tiab]) OR (feasibility trial[tiab]) OR (phase I trial[tiab]) OR (phase II trial[tiab]) OR (phase III trial[tiab]) OR (phase IV trial[tiab]) OR (multicenter trial[tiab]) OR (single -center trial[tiab]) OR (international trial[tiab]) OR (national trial[tiab]) OR (regional trial[tiab])
7	(inflammatory response[tiab]) OR (inflammation[tiab]) OR (inflammatory reaction[tiab]) OR (inflammatory process[tiab]) OR (inflammatory cascade[tiab]) OR (inflammatory activation[tiab]) OR (pro -inflammatory response[tiab]) OR (pro -inflammatory reaction[tiab]) OR (pro - inflammatory process[tiab]) OR (systemic inflammatory response[tiab]) OR (SIRS[tiab]) OR (local inflammatory response[tiab]) OR (acute inflammatory response[tiab]) OR (chronic inflammatory response[tiab]) OR (inflammatory mediator release[tiab]) OR (cytokine release[tiab]) OR (chemokine release[tiab]) OR (inflammatory cell activation[tiab]) OR (leukocyte activation[tiab]) OR (macrophage activation[tiab]) OR (neutrophil activation[tiab]) OR (inflammatory signaling[tiab]) OR (inflammatory pathway activation[tiab]) OR (inflammatory storm[tiab]) OR (cytokine storm[tiab]) OR (inflammatory cytokine network[tiab]) OR (inflammatory mediator storm[tiab]) OR (inflammatory cell recruitment[tiab]) OR (inflammatory cell infiltration[tiab]) OR (inflammatory cell migration[tiab]) OR (inflammatory cell adhesion[tiab]) OR (inflammatory cell extravasation[tiab]) OR (inflammatory cell transmigration[tiab])
6	(organ dysfunction[tiab]) OR (organ failure[tiab]) OR (organ insufficiency[tiab]) OR (organ damage[tiab]) OR (organ injury[tiab]) OR (organ impairment[tiab]) OR (organ lesion[tiab]) OR (organ defect[tiab]) OR (organ abnormality[tiab]) OR (organ disorder[tiab]) OR (organ malfunction[tiab]) OR (organ non -function[tiab]) OR (multi -organ dysfunction[tiab]) OR (MOD[tiab]) OR (multi -organ failure[tiab]) OR (MOF[tiab]) OR (acute organ dysfunction[tiab]) OR (chronic organ dysfunction[tiab]) OR (systemic organ dysfunction[tiab]) OR (organ -specific dysfunction[tiab]) OR (organ -based dysfunction[tiab]) OR (organ -related dysfunction[tiab]) OR (renal dysfunction[tiab]) OR (renal failure[tiab]) OR (hepatic dysfunction[tiab]) OR (hepatic failure[tiab]) OR (pulmonary dysfunction[tiab]) OR (pulmonary failure[tiab]) OR (cardiac dysfunction[tiab]) OR (cardiac failure[tiab]) OR (neurological dysfunction[tiab]) OR

	(neurological failure[tiab]) OR (gastrointestinal dysfunction[tiab]) OR (gastrointestinal failure[tiab]) OR (hematological dysfunction[tiab]) OR (hematological failure[tiab]) OR (respiratory dysfunction[tiab]) OR (respiratory failure[tiab]) OR (cardiopulmonary dysfunction[tiab]) OR (cardiopulmonary failure[tiab]) OR (neurocognitive dysfunction[tiab]) OR (neurocognitive failure[tiab]) OR (metabolic dysfunction[tiab]) OR (metabolic failure[tiab]) OR (endocrine dysfunction[tiab]) OR (endocrine failure[tiab]) OR (musculoskeletal dysfunction[tiab]) OR (musculoskeletal failure[tiab])
5	(survival rate[tiab]) OR (survival rates[tiab]) OR (survival ratio[tiab]) OR (survival ratios[tiab]) OR (survival percentage[tiab]) OR (survival percentages[tiab]) OR (survival proportion[tiab]) OR (survival proportions[tiab]) OR (mortality rate[tiab]) OR (mortality rates[tiab]) OR (death rate[tiab]) OR (death rates[tiab]) OR (fatality rate[tiab]) OR (fatality rates[tiab]) OR (lethality rate[tiab]) OR (lethality rates[tiab]) OR (case -fatality rate[tiab]) OR (case -fatality rates[tiab]) OR (survival probability[tiab]) OR (survival probabilities[tiab]) OR (survival odds[tiab]) OR (survival chances[tiab]) OR (survival likelihood[tiab]) OR (patient survival[tiab]) OR (long -term survival[tiab]) OR (short -term survival[tiab]) OR (overall survival[tiab]) OR (disease -specific survival[tiab]) OR (cause -specific survival[tiab]) OR (event -free survival[tiab]) OR (relapse -free survival[tiab]) OR (progression -free survival[tiab]) OR (metastasis -free survival[tiab]) OR (survival time[tiab]) OR (time to death[tiab]) OR (time to event[tiab]) OR (life expectancy[tiab]) OR (life expectancies[tiab]) OR (prognosis[tiab]) OR (prognoses[tiab]) OR (predictive survival[tiab]) OR (survival prediction[tiab]) OR (survival prognosis[tiab]) OR (survival estimation[tiab]) OR (survival assessment[tiab]) OR (survival analysis[tiab]) OR (survival monitoring[tiab])
4	(therapeutic effects[tiab]) OR (therapeutic efficacy[tiab]) OR (therapeutic benefits[tiab]) OR (treatment effects[tiab]) OR (treatment efficacy[tiab]) OR (treatment benefits[tiab]) OR (therapeutic outcomes[tiab]) OR (treatment outcomes[tiab]) OR (therapeutic responses[tiab]) OR (treatment responses[tiab]) OR (therapeutic success[tiab]) OR (treatment success[tiab]) OR (therapeutic improvement[tiab]) OR (treatment improvement[tiab]) OR (therapeutic advantages[tiab]) OR (treatment advantages[tiab]) OR (therapeutic gains[tiab]) OR (treatment gains[tiab]) OR (therapeutic value[tiab]) OR (treatment value[tiab]) OR (therapeutic potentials[tiab]) OR (treatment potentials[tiab]) OR (healing effects[tiab]) OR (remedial effects[tiab]) OR (curative effects[tiab]) OR (recovery effects[tiab]) OR (restorative effects[tiab]) OR (repair effects[tiab]) OR (regenerative effects[tiab]) OR (ameliorative effects[tiab]) OR (mitigative effects[tiab]) OR (alleviative effects[tiab]) OR (therapeutic effectiveness[tiab]) OR (therapeutic effectivity[tiab]) OR (therapeutic profit[tiab]) OR (therapeutic achievement[tiab]) OR (therapeutic accomplishment[tiab]) OR (therapeutic achievement[tiab]) OR (therapeutic achievement[tiab]) OR (therapeutic achievement[tiab])
3	(extracellular vesicles[tiab]) OR (exosomes[tiab]) OR (microvesicles[tiab]) OR (microparticles[tiab]) OR (vesicles, extracellular[tiab]) OR (exosome -like vesicles[tiab]) OR (exosome -like particles[tiab]) OR (small extracellular vesicles[tiab]) OR (sEVs[tiab]) OR (large extracellular vesicles[tiab]) OR (lEVs[tiab]) OR (ectosomes[tiab]) OR (exosome -derived vesicles[tiab]) OR (exosome -secreted vesicles[tiab]) OR (exosome -containing vesicles[tiab]) OR (exosome -enriched vesicles[tiab]) OR (exosome -mediated vesicles[tiab]) OR (vesicle -mediated signaling in sepsis[tiab]) OR (exosome -based therapy for sepsis[tiab]) OR (extracellular vesicle -based therapy for sepsis[tiab]) OR (exosome -mediated immune modulation in sepsis[tiab]) OR

	(exosome -induced inflammation in sepsis[tiab]) OR (exosome -mediated cell -cell communication in sepsis[tiab]) OR (cell -derived vesicles in sepsis[tiab]) OR (cell -secreted vesicles in sepsis[tiab]) OR (vesicle -mediated immune response in sepsis[tiab]) OR (exosome -mediated cytokine release in sepsis[tiab]) OR (exosome -mediated chemokine release in sepsis[tiab]) OR (exosome -mediated leukocyte recruitment in sepsis[tiab]) OR (exosome -mediated endothelial activation in sepsis[tiab]) OR (exosome -mediated coagulation in sepsis[tiab])
2	(mesenchymal stem cells[tiab]) OR (MSCs[tiab]) OR (multipotent stromal cells[tiab]) OR (stromal vascular fraction[tiab]) OR (SVF[tiab]) OR (adipose -derived stem cells[tiab]) OR (ADSCs[tiab]) OR (bone marrow -derived mesenchymal stem cells[tiab]) OR (BM-MSCs[tiab]) OR (umbilical cord -derived mesenchymal stem cells[tiab]) OR (UC-MSCs[tiab]) OR (wharton's jelly -derived mesenchymal stem cells[tiab]) OR (WJ-MSCs[tiab]) OR (placenta -derived mesenchymal stem cells[tiab]) OR (PD-MSCs[tiab]) OR (amniotic fluid -derived mesenchymal stem cells[tiab]) OR (AF-MSCs[tiab]) OR (tooth pulp -derived mesenchymal stem cells[tiab]) OR (menstrual blood -derived mesenchymal stem cells[tiab]) OR (MB-MSCs[tiab]) OR (tissue -specific mesenchymal stem cells[tiab]) OR (TS-MSCs[tiab]) OR (multipotent mesenchymal stromal cells[tiab]) OR (MMSCs[tiab]) OR (mesenchymal stem cell therapy[tiab]) OR (MSC therapy[tiab]) OR (mesenchymal stem cell -based therapy[tiab]) OR (MSC -based therapy[tiab]) OR (regenerative therapy, mesenchymal stem cells[tiab]) OR (regenerative therapy, MSCs[tiab]) OR (cell -based therapy, mesenchymal stem cells[tiab]) OR (cell -based therapy, MSCs[tiab])
1	(sepsis[tiab]) OR (septic shock[tiab]) OR (severe sepsis[tiab]) OR (blood poisoning[tiab]) OR (septicemia[tiab]) OR (systemic inflammatory response syndrome due to infection[tiab]) OR (SIRS due to infection[tiab]) OR (sepsis syndrome[tiab]) OR (septic infection[tiab]) OR (sepsis and septic shock[tiab]) OR (severe sepsis and septic shock[tiab]) OR (sepsis -related[tiab]) OR (sepsis-induced[tiab]) OR (sepsis-associated[tiab]) OR (sepsis-triggered[tiab]) OR (sepsis-mediated[tiab]) OR (bacteremia with sepsis[tiab]) OR (infectious sepsis[tiab]) OR (sepsis, organ dysfunction[tiab]) OR (sepsis, multiorgan failure[tiab]) OR (sepsis, DIC[tiab]) OR (sepsis, disseminated intravascular coagulation[tiab]) OR (sepsis, acute respiratory distress syndrome[tiab]) OR (ARDS in sepsis[tiab]) OR (sepsis with acute kidney injury[tiab]) OR (AKI in sepsis[tiab]) OR (sepsis, septic encephalopathy[tiab]) OR (septic encephalopathy in sepsis[tiab]) OR (sepsis, myocardial depression[tiab]) OR (myocardial depression in sepsis[tiab]) OR (postoperative sepsis[tiab]) OR (trauma -induced sepsis[tiab]) OR (burn -induced sepsis[tiab]) OR (neonatal sepsis[tiab]) OR (pediatric sepsis[tiab]) OR (geriatric sepsis[tiab])

Web of Science

10	#8 AND #1 AND (#4 OR #6 OR #7) AND (#2 OR #3) AND #5
9	#1 AND (#4 OR #6 OR #7) AND (#2 OR #3) AND #5
8	TS=(controlled clinical trial OR randomized controlled trial OR randomized OR drug therapy OR trial OR drug therapy OR randomly OR groups OR RCT OR randomized trial OR random allocation OR random assignment OR placebo-controlled trial OR double-blind trial OR single-blind trial OR open-label trial OR parallel-group trial OR crossover trial OR pharmacotherapy OR medication OR treatment OR intervention OR therapeutic trial OR clinical study OR research trial OR treatment group OR control group OR prospective study OR retrospective study OR observational study OR cohort study OR case-control study OR

	cross-sectional study OR longitudinal study OR interventional study OR non-randomized trial OR quasi-experimental trial OR pilot trial OR feasibility trial OR phase I trial OR phase II trial OR phase III trial OR phase IV trial OR multicenter trial OR single-center trial OR international trial OR national trial OR regional trial)
7	TS=(inflammatory response OR inflammation OR inflammatory reaction OR inflammatory process OR inflammatory cascade OR inflammatory activation OR pro-inflammatory response OR pro-inflammatory reaction OR pro-inflammatory process OR systemic inflammatory response OR SIRS OR local inflammatory response OR acute inflammatory response OR chronic inflammatory response OR inflammatory mediator release OR cytokine release OR chemokine release OR inflammatory cell activation OR leukocyte activation OR macrophage activation OR neutrophil activation OR inflammatory signaling OR inflammatory pathway activation OR inflammatory storm OR cytokine storm OR inflammatory cytokine network OR inflammatory mediator storm OR inflammatory cell recruitment OR inflammatory cell infiltration OR inflammatory cell migration OR inflammatory cell adhesion OR inflammatory cell extravasation OR inflammatory cell transmigration)
6	TS=(organ dysfunction OR organ failure OR organ insufficiency OR organ damage OR organ injury OR organ impairment OR organ lesion OR organ defect OR organ abnormality OR organ disorder OR organ malfunction OR organ non-function OR multi-organ dysfunction OR MOD OR multi-organ failure OR MOF OR acute organ dysfunction OR chronic organ dysfunction OR systemic organ dysfunction OR organ-specific dysfunction OR organ-based dysfunction OR organ-related dysfunction OR renal dysfunction OR renal failure OR hepatic dysfunction OR hepatic failure OR pulmonary dysfunction OR pulmonary failure OR cardiac dysfunction OR cardiac failure OR neurological dysfunction OR neurological failure OR gastrointestinal dysfunction OR gastrointestinal failure OR hematological dysfunction OR hematological failure OR respiratory dysfunction OR respiratory failure OR cardiopulmonary dysfunction OR cardiopulmonary failure OR neurocognitive dysfunction OR neurocognitive failure OR metabolic dysfunction OR metabolic failure OR endocrine dysfunction OR endocrine failure OR musculoskeletal dysfunction OR musculoskeletal failure)
5	TS=(survival rate OR survival rates OR survival ratio OR survival ratios OR survival percentage OR survival percentages OR survival proportion OR survival proportions OR mortality rate OR mortality rates OR death rate OR death rates OR fatality rate OR fatality rates OR lethality rate OR lethality rates OR case-fatality rate OR case-fatality rates OR survival probability OR survival probabilities OR survival odds OR survival chances OR survival likelihood OR patient survival OR long-term survival OR short-term survival OR overall survival OR disease-specific survival OR cause-specific survival OR event-free survival OR relapse-free survival OR progression-free survival OR metastasis-free survival OR survival time OR time to death OR time to event OR life expectancy OR life expectancies OR prognosis OR prognoses OR predictive survival OR survival prediction OR survival prognosis OR survival estimation OR survival assessment OR survival analysis OR survival monitoring)
4	TS=(therapeutic effects OR therapeutic efficacy OR therapeutic benefits OR treatment effects OR treatment efficacy OR treatment benefits OR therapeutic outcomes OR treatment outcomes OR therapeutic responses OR treatment responses OR therapeutic success OR

	treatment success OR therapeutic improvement OR treatment improvement OR therapeutic advantages OR treatment advantages OR therapeutic gains OR treatment gains OR therapeutic value OR treatment value OR therapeutic potentials OR treatment potentials OR healing effects OR remedial effects OR curative effects OR recovery effects OR restorative effects OR repair effects OR regenerative effects OR ameliorative effects OR mitigative effects OR alleviative effects OR therapeutic effectiveness OR therapeutic effectivity OR therapeutic profit OR therapeutic achievement OR therapeutic accomplishment OR therapeutic achievement OR therapeutic achievement OR therapeutic achievement)
3	TS=(extracellular vesicles OR exosomes OR microvesicles OR microparticles OR vesicles, extracellular OR exosome-like vesicles OR exosome-like particles OR small extracellular vesicles OR sEVs OR large extracellular vesicles OR lEVs OR ectosomes OR exosome-derived vesicles OR exosome-secreted vesicles OR exosome-containing vesicles OR exosome-enriched vesicles OR exosome-mediated vesicles OR vesicle-mediated signaling in sepsis OR exosome-based therapy for sepsis OR extracellular vesicle-based therapy for sepsis OR exosome-mediated immune modulation in sepsis OR exosome-induced inflammation in sepsis OR exosome-mediated cell-cell communication in sepsis OR cell-derived vesicles in sepsis OR cell-secreted vesicles in sepsis OR vesicle-mediated immune response in sepsis OR exosome-mediated cytokine release in sepsis OR exosome-mediated chemokine release in sepsis OR exosome-mediated leukocyte recruitment in sepsis OR exosome-mediated endothelial activation in sepsis OR exosome-mediated coagulation in sepsis)
2	TS=(mesenchymal stem cells OR MSCs OR multipotent stromal cells OR stromal vascular fraction OR SVF OR adipose-derived stem cells OR ADSCs OR bone marrow-derived mesenchymal stem cells OR BM-MSCs OR umbilical cord-derived mesenchymal stem cells OR UC-MSCs OR wharton's jelly-derived mesenchymal stem cells OR WJ-MSCs OR placenta-derived mesenchymal stem cells OR PD-MSCs OR amniotic fluid-derived mesenchymal stem cells OR AF-MSCs OR tooth pulp-derived mesenchymal stem cells OR menstrual blood-derived mesenchymal stem cells OR MB-MSCs OR tissue-specific mesenchymal stem cells OR TS-MSCs OR multipotent mesenchymal stromal cells OR MMSCs OR mesenchymal stem cell therapy OR MSC therapy OR mesenchymal stem cell-based therapy OR MSC-based therapy OR regenerative therapy, mesenchymal stem cells OR regenerative therapy, MSCs OR cell-based therapy, mesenchymal stem cells OR cell-based therapy, MSCs)
1	TS=(sepsis OR septic shock OR severe sepsis OR blood poisoningOR septicemia OR systemic inflammatory response syndrome due to infection OR SIRS due to infection OR sepsis syndrome OR septic infection OR sepsis and septic shock OR severe sepsis and septic shockOR sepsis-related OR sepsis-induced OR sepsis-associated OR sepsis-triggered OR sepsis-mediated OR bacteremia with sepsis OR infectious sepsis OR sepsis, organ dysfunction OR sepsis, multiorgan failure OR sepsis, DIC OR sepsis, disseminated intravascular coagulation OR sepsis, acute respiratory distress syndrome OR ARDS in sepsis OR sepsis with acute kidney injury OR AKI in sepsis OR sepsis, septic encephalopathy OR septic encephalopathy in sepsis OR sepsis, myocardial depression OR myocardial depression in sepsis OR postoperative sepsis OR trauma -induced sepsis OR burn-induced sepsis OR neonatal sepsis OR pediatric sepsis OR geriatric sepsis)

Embase

10	#8 AND #1 AND (#4 OR #6 OR #7) AND (#2 OR #3) AND #5
9	#1 AND (#4 OR #6 OR #7) AND (#2 OR #3) AND #5
8	controlled:pt AND clinical:pt AND trial:pt OR (randomized:pt AND controlled:pt AND trial:pt) OR ((randomized:ti,ab OR drug:ti,ab) AND therapy:ti,ab) OR randomly:ti,ab OR trial:ti,ab OR groups:ti,ab OR rct:ti,ab OR (randomized:ti,ab AND trial:ti,ab) OR (random:ti,ab AND allocation:ti,ab) OR (random:ti,ab AND assignment:ti,ab) OR (placebo:ti,ab AND -controlled:ti,ab AND trial:ti,ab) OR (double:ti,ab AND -blind:ti,ab AND trial:ti,ab) OR (single:ti,ab AND -blind:ti,ab AND trial:ti,ab) OR (open:ti,ab AND -label:ti,ab AND trial:ti,ab) OR (parallel:ti,ab AND -group:ti,ab AND trial:ti,ab) OR (crossover:ti,ab AND trial:ti,ab) OR pharmacotherapy:ti,ab OR medication:ti,ab OR treatment:ti,ab OR intervention:ti,ab OR (therapeutic:ti,ab AND trial:ti,ab) OR (clinical:ti,ab AND study:ti,ab) OR (research:ti,ab AND trial:ti,ab) OR (treatment:ti,ab AND group:ti,ab) OR (control:ti,ab AND group:ti,ab) OR (prospective:ti,ab AND study:ti,ab) OR (retrospective:ti,ab AND study:ti,ab) OR (observational:ti,ab AND study:ti,ab) OR (cohort:ti,ab AND study:ti,ab) OR (case:ti,ab AND -control:ti,ab AND study:ti,ab) OR (cross:ti,ab AND -sectional:ti,ab AND study:ti,ab) OR (longitudinal:ti,ab AND study:ti,ab) OR (interventional:ti,ab AND study:ti,ab) OR (non:ti,ab AND -randomized:ti,ab AND trial:ti,ab) OR (quasi:ti,ab AND -experimental:ti,ab AND trial:ti,ab) OR (pilot:ti,ab AND trial:ti,ab) OR (feasibility:ti,ab AND trial:ti,ab) OR (phase:ti,ab AND i:ti,ab AND trial:ti,ab) OR (phase:ti,ab AND ii:ti,ab AND trial:ti,ab) OR (phase:ti,ab AND iii:ti,ab AND trial:ti,ab) OR (phase:ti,ab AND iv:ti,ab AND trial:ti,ab) OR (multicenter:ti,ab AND trial:ti,ab) OR (single:ti,ab AND -center:ti,ab AND trial:ti,ab) OR (international:ti,ab AND trial:ti,ab) OR (national:ti,ab AND trial:ti,ab) OR (regional:ti,ab AND trial:ti,ab)
7	'therapeutic effects' OR 'therapeutic efficacy'/exp OR 'therapeutic benefits' OR 'treatment effects' OR 'treatment efficacy'/exp OR 'treatment benefits' OR 'therapeutic outcomes' OR 'treatment outcomes' OR 'therapeutic responses' OR 'treatment responses' OR 'therapeutic success' OR 'treatment success'/exp OR 'therapeutic improvement' OR 'treatment improvement' OR 'therapeutic advantages' OR 'treatment advantages' OR 'therapeutic gains' OR 'treatment gains' OR 'therapeutic value'/exp OR 'treatment value' OR 'therapeutic potentials' OR 'treatment potentials' OR 'healing effects' OR 'remedial effects' OR 'curative effects' OR 'recovery effects' OR 'restorative effects' OR 'repair effects' OR 'regenerative effects' OR 'ameliorative effects' OR 'mitigative effects' OR 'alleviative effects' OR 'therapeutic effectiveness' OR 'therapeutic effectivity' OR 'therapeutic profit' OR 'therapeutic achievement' OR 'therapeutic accomplishment'
6	'survival rate'/exp OR 'survival rates' OR 'survival ratio' OR 'survival ratios' OR 'survival percentage' OR 'survival percentages' OR 'survival proportion' OR 'survival proportions' OR 'mortality rate'/exp OR 'mortality rates' OR 'death rate'/exp OR 'death rates' OR 'fatality rate'/exp OR 'fatality rates' OR 'lethality rate'/exp OR 'lethality rates' OR 'case -fatality rate'/exp OR 'case -fatality rates' OR 'survival probability'/exp OR 'survival probabilities' OR 'survival odds' OR 'survival chances' OR 'survival likelihood' OR 'patient survival'/exp OR 'long -term survival'/exp OR 'short -term survival'/exp OR 'overall survival'/exp OR 'disease -specific survival'/exp OR 'cause -specific survival'/exp OR 'event -free survival'/exp OR 'relapse -free survival'/exp OR 'progression -free survival'/exp OR 'metastasis -free survival'/exp OR 'survival time'/exp OR 'time to death'/exp OR 'time to event'/exp OR 'life expectancy'/exp OR 'life expectancies' OR 'prognosis'/exp OR 'prognoses' OR 'predictive survival' OR 'survival prediction'/exp OR 'survival prognosis' OR 'survival estimation' OR 'survival assessment' OR

	'survival analysis'/exp OR 'survival monitoring'
5	'organ dysfunction'/exp OR 'organ failure'/exp OR 'organ insufficiency' OR 'organ damage'/exp OR 'organ injury'/exp OR 'organ impairment' OR 'organ lesion' OR 'organ defect' OR 'organ abnormality' OR 'organ disorder' OR 'organ malfunction' OR 'organ non -function' OR 'multi -organ dysfunction' OR 'mod' OR 'multi -organ failure' OR 'mof' OR 'acute organ dysfunction' OR 'chronic organ dysfunction' OR 'systemic organ dysfunction' OR 'organ -specific dysfunction' OR 'organ -based dysfunction' OR 'organ -related dysfunction' OR 'renal dysfunction'/exp OR 'renal failure'/exp OR 'hepatic dysfunction'/exp OR 'hepatic failure'/exp OR 'pulmonary dysfunction'/exp OR 'pulmonary failure'/exp OR 'cardiac dysfunction'/exp OR 'cardiac failure'/exp OR 'neurological dysfunction' OR 'neurological failure' OR 'gastrointestinal dysfunction'/exp OR 'gastrointestinal failure'/exp OR 'hematological dysfunction' OR 'hematological failure' OR 'respiratory dysfunction'/exp OR 'respiratory failure'/exp OR 'cardiopulmonary dysfunction'/exp OR 'cardiopulmonary failure'/exp OR 'neurocognitive dysfunction'/exp OR 'neurocognitive failure' OR 'metabolic dysfunction'/exp OR 'metabolic failure' OR 'endocrine dysfunction'/exp OR 'endocrine failure' OR 'musculoskeletal dysfunction' OR 'musculoskeletal failure'
4	'inflammatory response'/exp OR 'inflammation'/exp OR 'inflammatory reaction'/exp OR 'inflammatory process'/exp OR 'inflammatory cascade' OR 'inflammatory activation' OR 'pro -inflammatory response' OR 'pro -inflammatory reaction' OR 'pro -inflammatory process' OR 'systemic inflammatory response'/exp OR 'sirs' OR 'local inflammatory response' OR 'acute inflammatory response' OR 'chronic inflammatory response' OR 'inflammatory mediator release' OR 'cytokine release'/exp OR 'chemokine release' OR 'inflammatory cell activation' OR 'leukocyte activation'/exp OR 'macrophage activation'/exp OR 'neutrophil activation'/exp OR 'inflammatory signaling'/exp OR 'inflammatory pathway activation' OR 'inflammatory storm' OR 'cytokine storm'/exp OR 'inflammatory cytokine network' OR 'inflammatory mediator storm' OR 'inflammatory cell recruitment' OR 'inflammatory cell infiltration'/exp OR 'inflammatory cell migration' OR 'inflammatory cell adhesion' OR 'inflammatory cell extravasation' OR 'inflammatory cell transmigration'
3	'extracellular vesicles'/exp OR 'exosomes'/exp OR 'microvesicles' OR 'microparticles'/exp OR 'vesicles, extracellular' OR 'exosome -like vesicles' OR 'exosome -like particles' OR 'small extracellular vesicles' OR 'sevs' OR 'large extracellular vesicles' OR 'levs' OR 'ectosomes' OR 'exosome -derived vesicles' OR 'exosome -secreted vesicles' OR 'exosome -containing vesicles' OR 'exosome -enriched vesicles' OR 'exosome -mediated vesicles' OR 'vesicle -mediated signaling in sepsis' OR 'exosome -based therapy for sepsis' OR 'extracellular vesicle -based therapy for sepsis' OR 'exosome -mediated immune modulation in sepsis' OR 'exosome -induced inflammation in sepsis' OR 'exosome -mediated cell -cell communication in sepsis' OR 'cell -derived vesicles in sepsis' OR 'cell -secreted vesicles in sepsis' OR 'vesicle -mediated immune response in sepsis' OR 'exosome -mediated cytokine release in sepsis' OR 'exosome -mediated chemokine release in sepsis' OR 'exosome -mediated leukocyte recruitment in sepsis' OR 'exosome -mediated endothelial activation in sepsis' OR 'exosome -mediated coagulation in sepsis'
2	((((((((((((((((((mesenchymal AND stem AND 'cells'/exp OR mscs OR multipotent) AND stromal AND 'cells'/exp OR stromal) AND vascular AND fraction OR svf OR adipose) AND -derived AND stem AND 'cells'/exp OR adscs OR bone) AND marrow AND -derived AND mesenchymal AND stem AND 'cells'/exp OR 'bm mscs' OR umbilical) AND cord AND -derived AND mesenchymal AND stem AND 'cells'/exp OR 'uc mscs' OR whartons) AND jelly AND -derived AND mesenchymal AND stem AND 'cells'/exp OR 'wj mscs' OR placenta) AND -derived AND mesenchymal AND stem AND

	'cells'/exp OR 'pd mscs' OR amniotic) AND fluid AND -derived AND mesenchymal AND stem AND 'cells'/exp OR 'af mscs' OR tooth) AND pulp AND -derived AND mesenchymal AND stem AND 'cells'/exp OR menstrual) AND blood AND -derived AND mesenchymal AND stem AND 'cells'/exp OR 'mb mscs' OR tissue) AND -specific AND mesenchymal AND stem AND 'cells'/exp OR 'ts mscs' OR multipotent) AND mesenchymal AND stromal AND 'cells'/exp OR mmscs OR mesenchymal) AND stem AND cell AND 'therapy'/exp OR msc) AND 'therapy'/exp OR mesenchymal) AND stem AND cell AND -based AND 'therapy'/exp OR msc) AND -based AND 'therapy'/exp OR regenerative) AND therapy, AND mesenchymal AND stem AND 'cells'/exp OR regenerative) AND therapy, AND mscs OR cell) AND -based AND therapy, AND mesenchymal AND stem AND 'cells'/exp OR cell) AND -based AND therapy, AND mscs
1	'sepsis'/exp OR 'septic shock'/exp OR 'severe sepsis'/exp OR 'blood poisoning' OR 'septicemia'/exp OR 'systemic inflammatory response syndrome due to infection' OR 'sirs due to infection' OR 'sepsis syndrome'/exp OR 'septic infection' OR 'sepsis and septic shock' OR 'severe sepsis and septic shock' OR 'sepsis -related' OR 'sepsis-induced' OR 'sepsis-associated' OR 'sepsis-triggered' OR 'sepsis-mediated' OR 'bacteremia with sepsis' OR 'infectious sepsis' OR 'sepsis, organ dysfunction' OR 'sepsis, multiorgan failure' OR 'sepsis, dic' OR 'sepsis, disseminated intravascular coagulation' OR 'sepsis, acute respiratory distress syndrome' OR 'ards in sepsis' OR 'sepsis with acute kidney injury' OR 'aki in sepsis' OR 'sepsis, septic encephalopathy' OR 'septic encephalopathy in sepsis' OR 'sepsis, myocardial depression' OR 'myocardial depression in sepsis' OR 'postoperative sepsis'/exp OR 'trauma -induced sepsis' OR 'burn -induced sepsis' OR 'neonatal sepsis'/exp OR 'pediatric sepsis'/exp OR 'geriatric sepsis'

Cochrane Library

14	#12 AND #13
13	(#1 OR #6) AND #2 AND (#3 OR #7 OR #8) AND (#9 OR #10)
12	#11 NOT (#4 NOT #5)
11	(controlled clinical trial):pt OR (randomized controlled trial):pt OR (randomized OR drug therapy OR randomly OR trial OR groups)
10	(survival rate OR survival rates OR survival ratio OR survival ratios OR survival percentage OR survival percentages OR survival proportion OR survival proportions OR mortality rate OR mortality rates OR death rate OR death rates OR fatality rate OR fatality rates OR lethality rate OR lethality rates OR case-fatality rate OR case-fatality rates OR survival probability OR survival probabilities OR survival odds OR survival chances OR survival likelihood OR patient survival OR long-term survival OR short-term survival OR overall survival OR disease-specific survival OR cause-specific survival OR event-free survival OR relapse-free survival OR progression-free survival OR metastasis-free survival OR survival time OR time to death OR time to event OR life expectancy OR life expectancies OR prognosis OR prognoses OR predictive survival OR survival prediction OR survival prognosis OR survival estimation OR survival assessment OR survival analysis OR survival monitoring):ti,ab,kw (Word variations have been searched)
9	(therapeutic effects OR therapeutic efficacy OR therapeutic benefits OR treatment effects OR treatment efficacy OR treatment benefits OR therapeutic outcomes OR treatment outcomes OR therapeutic responses OR treatment responses OR therapeutic success OR treatment success OR therapeutic improvement OR treatment improvement OR therapeutic advantages OR treatment advantages OR therapeutic gains OR treatment gains OR therapeutic value OR treatment value OR therapeutic potentials

	OR treatment potentials OR healing effects OR remedial effects OR curative effects OR recovery effects OR restorative effects OR repair effects OR regenerative effects OR ameliorative effects OR mitigative effects OR alleviative effects OR therapeutic effectiveness OR therapeutic effectivity OR therapeutic profit OR therapeutic achievement OR therapeutic accomplishment):ti,ab,kw (Word variations have been searched)
8	(extracellular vesicles OR exosomes OR microvesicles OR microparticles OR vesicles, extracellular OR exosome-like vesicles OR exosome-like particles OR small extracellular vesicles OR sEVs OR large extracellular vesicles OR lEVs OR ectosomes OR exosome-derived vesicles OR exosome-secreted vesicles OR exosome-containing vesicles OR exosome-enriched vesicles OR exosome-mediated vesicles OR vesicle-mediated signaling in sepsis OR exosome-based therapy for sepsis OR extracellular vesicle-based therapy for sepsis OR exosome-mediated immune modulation in sepsis OR exosome-induced inflammation in sepsis OR exosome-mediated cell-cell communication in sepsis OR cell-derived vesicles in sepsis OR cell-secreted vesicles in sepsis OR vesicle-mediated immune response in sepsis OR exosome-mediated cytokine release in sepsis OR exosome-mediated chemokine release in sepsis OR exosome-mediated leukocyte recruitment in sepsis OR exosome-mediated endothelial activation in sepsis OR exosome-mediated coagulation in sepsis):ti,ab,kw (Word variations have been searched)
7	(mesenchymal stem cells OR MSCs OR multipotent stromal cells OR stromal vascular fraction OR SVF OR adipose-derived stem cells OR ADSCs OR bone marrow-derived mesenchymal stem cells OR BM-MSCs OR umbilical cord-derived mesenchymal stem cells OR UC-MSCs OR wharton's jelly-derived mesenchymal stem cells OR WJ-MSCs OR placenta-derived mesenchymal stem cells OR PD-MSCs OR amniotic fluid-derived mesenchymal stem cells OR AF-MSCs OR tooth pulp-derived mesenchymal stem cells OR menstrual blood-derived mesenchymal stem cells OR MB-MSCs OR tissue-specific mesenchymal stem cells OR TS-MSCs OR multipotent mesenchymal stromal cells OR MMSCs OR mesenchymal stem cell therapy OR MSC therapy OR mesenchymal stem cell-based therapy OR MSC-based therapy OR regenerative therapy, mesenchymal stem cells OR regenerative therapy, MSCs OR cell-based therapy, mesenchymal stem cells OR cell-based therapy, MSCs):ti,ab,kw (Word variations have been searched)
6	(sepsis OR septic shock OR severe sepsis OR blood poisoning OR septicemia OR "systemic inflammatory response syndrome due to infection" OR SIRS OR sepsis syndrome OR septic infection OR sepsis-related OR sepsis-induced OR sepsis-associated OR sepsis-triggered OR sepsis-mediated OR bacteremia OR infectious sepsis OR "organ dysfunction" OR "multiorgan failure" OR DIC OR "disseminated intravascular coagulation" OR ARDS OR "acute respiratory distress syndrome" OR AKI OR "acute kidney injury" OR "septic encephalopathy" OR "myocardial depression" OR postoperative sepsis OR "trauma-induced sepsis" OR "burn-induced sepsis" OR neonatal sepsis OR pediatric sepsis OR geriatric sepsis):ti,ab,kw (Word variations have been searched):ti,ab,kw (Word variations have been searched)
5	MeSH descriptor: [Humans] explode all trees
4	MeSH descriptor: [Animals] explode all trees
3	MeSH descriptor: [Exosomes] explode all trees
2	MeSH descriptor: [Liver Failure] explode all trees
1	MeSH descriptor: [Sepsis] explode all trees

Scopus

5	#1 AND #2 AND #3 AND #4

4	(TITLE-ABS-KEY("controlled clinical trial") OR TITLE-ABS-KEY("randomized controlled trial") OR TITLE-ABS-KEY("double-blind procedure") OR TITLE-ABS-KEY("single-blind procedure"))
3	(TITLE-ABS-KEY("sepsis") OR TITLE-ABS-KEY("septic shock") OR TITLE-ABS-KEY("severe sepsis") OR TITLE-ABS-KEY("blood poisoning") OR TITLE-ABS-KEY("septicemia") OR TITLE-ABS-KEY("systemic inflammatory response syndrome due to infection") OR TITLE-ABS-KEY("SIRS due to infection") OR TITLE-ABS-KEY("sepsis syndrome") OR TITLE-ABS-KEY("septic infection") OR TITLE-ABS-KEY("sepsis and septic shock") OR TITLE-ABS-KEY("severe sepsis and septic shock") OR TITLE-ABS-KEY("sepsis-related") OR TITLE-ABS-KEY("sepsis-induced") OR TITLE-ABS-KEY("sepsis-associated") OR TITLE-ABS-KEY("sepsis-triggered") OR TITLE-ABS-KEY("sepsis-mediated") OR TITLE-ABS-KEY("bacteremia with sepsis") OR TITLE-ABS-KEY("infectious sepsis") OR TITLE-ABS-KEY("sepsis, organ dysfunction") OR TITLE-ABS-KEY("sepsis, multiorgan failure") OR TITLE-ABS-KEY("sepsis, DIC") OR TITLE-ABS-KEY("sepsis, disseminated intravascular coagulation") OR TITLE-ABS-KEY("sepsis, acute respiratory distress syndrome") OR TITLE-ABS-KEY("ARDS in sepsis") OR TITLE-ABS-KEY("sepsis with acute kidney injury") OR TITLE-ABS-KEY("AKI in sepsis") OR TITLE-ABS-KEY("sepsis, septic encephalopathy") OR TITLE-ABS-KEY("septic encephalopathy in sepsis") OR TITLE-ABS-KEY("sepsis, myocardial depression") OR TITLE-ABS-KEY("myocardial depression in sepsis") OR TITLE-ABS-KEY("postoperative sepsis") OR TITLE-ABS-KEY("trauma-induced sepsis") OR TITLE-ABS-KEY("burn-induced sepsis") OR TITLE-ABS-KEY("neonatal sepsis") OR TITLE-ABS-KEY("pediatric sepsis") OR TITLE-ABS-KEY("geriatric sepsis"))
2	(TITLE-ABS-KEY("liver injury") OR TITLE-ABS-KEY("hepatic damage") OR TITLE-ABS-KEY("liver dysfunction") OR TITLE-ABS-KEY("hepatic failure") OR TITLE-ABS-KEY("acute liver failure") OR TITLE-ABS-KEY("chronic liver disease") OR TITLE-ABS-KEY("liver fibrosis") OR TITLE-ABS-KEY("hepatic fibrosis") OR TITLE-ABS-KEY("liver cirrhosis") OR TITLE-ABS-KEY("hepatic cirrhosis") OR TITLE-ABS-KEY("liver inflammation") OR TITLE-ABS-KEY("hepatic inflammation") OR TITLE-ABS-KEY("liver ischemia-reperfusion injury") OR TITLE-ABS-KEY("hepatic ischemia-reperfusion injury"))
1	(TITLE-ABS-KEY("extracellular vesicles") OR TITLE-ABS-KEY("exosomes") OR TITLE-ABS-KEY("microvesicles") OR TITLE-ABS-KEY("microparticles") OR TITLE-ABS-KEY("vesicles, extracellular") OR TITLE-ABS-KEY("exosome-like vesicles") OR TITLE-ABS-KEY("small extracellular vesicles") OR TITLE-ABS-KEY("sEVs") OR TITLE-ABS-KEY("large extracellular vesicles") OR TITLE-ABS-KEY("lEVs") OR TITLE-ABS-KEY("ectosomes") OR TITLE-ABS-KEY("exosome-derived vesicles") OR TITLE-ABS-KEY("exosome-secreted vesicles") OR TITLE-ABS-KEY("exosome-containing vesicles") OR TITLE-ABS-KEY("exosome-enriched vesicles") OR TITLE-ABS-KEY("vesicle-mediated signaling in sepsis") OR TITLE-ABS-KEY("exosome-based therapy for sepsis") OR TITLE-ABS-KEY("extracellular vesicle-based therapy for sepsis") OR TITLE-ABS-KEY("exosome-mediated immune modulation in sepsis") OR TITLE-ABS-KEY("exosome-induced inflammation in sepsis") OR TITLE-ABS-KEY("exosome-mediated cell-cell communication in sepsis") OR TITLE-ABS-KEY("cell-derived vesicles in sepsis") OR TITLE-ABS-KEY("cell-secreted vesicles in sepsis") OR TITLE-ABS-KEY("vesicle-mediated immune response in sepsis") OR TITLE-ABS-KEY("exosome-mediated cytokine release in sepsis") OR TITLE-ABS-KEY("exosome-mediated chemokine release in sepsis") OR TITLE-ABS-KEY("exosome-mediated leukocyte recruitment in sepsis") OR TITLE-ABS-KEY("exosome-mediated endothelial activation in sepsis") OR TITLE-ABS-KEY("exosome-mediated coagulation in sepsis"))

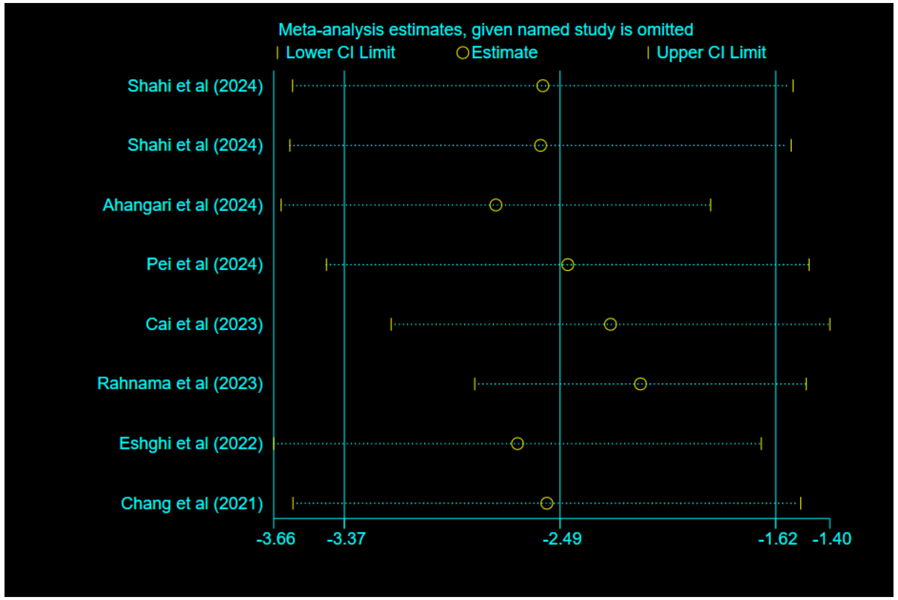

Supp Fig. 1. Sensitivity Analysis of MSC-sEVs on ALT

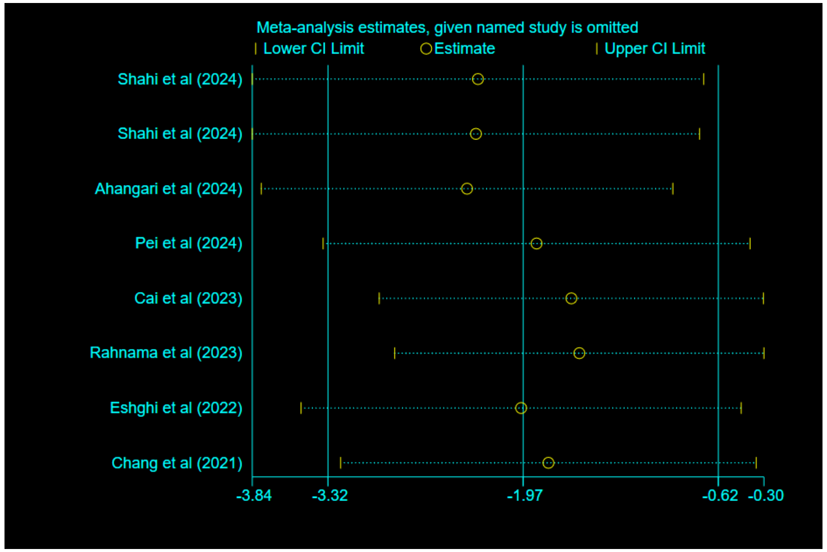

Supp Fig. 2. Sensitivity Analysis of MSC-sEVs on AST

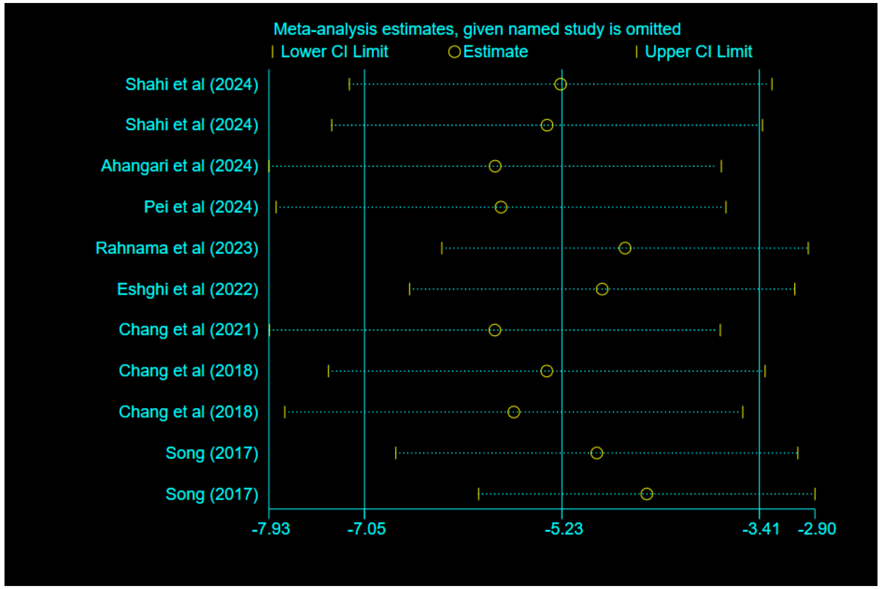

Supp Fig. 3. Sensitivity Analysis of MSC-sEVs on TNF-α

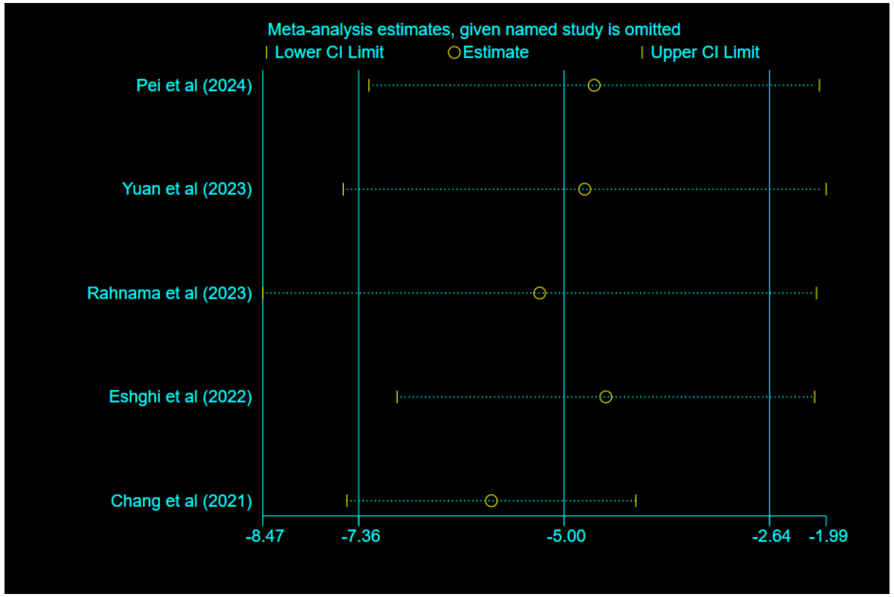

Meta-analysis estimates, given named study is omitted
Lower CI Limit
Estimate
Upper CI Limit
Pei et al (2024)
Yuan et al (2023)
Rahnama et al (2023)
Eshghi et al (2022)
Chang et al (2021)
-8.47
-7.36
-5.00
-2.64
-1.99

Supp Fig. 4. Sensitivity Analysis of MSC-sEVs on IL-6

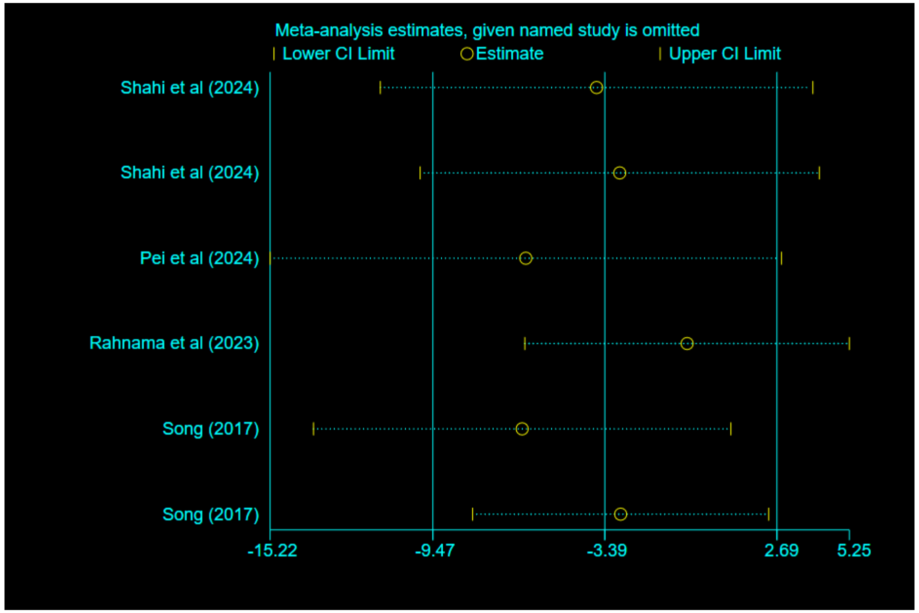

Supp Fig. 5. Sensitivity Analysis of MSC-sEVs on IL-10

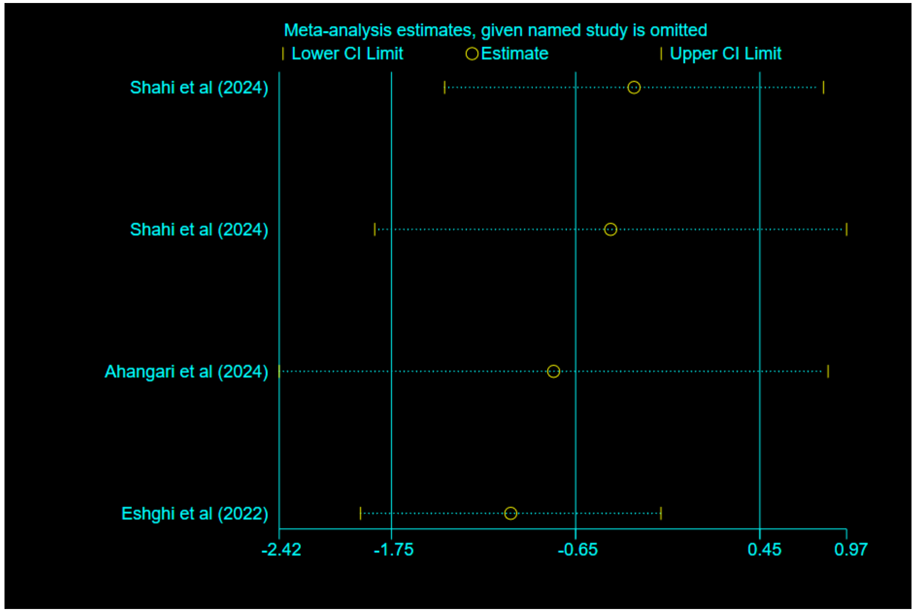

Meta-analysis estimates, given named study is omitted
Lower CI Limit
Estimate
Upper CI Limit
Shahi et al (2024)
Shahi et al (2024)
Ahangari et al (2024)
Eshghi et al (2022)
-2.42
-1.75
-0.65
0.45
0.97

Supp Fig. 6. Sensitivity Analysis of MSC-sEVs on NLR

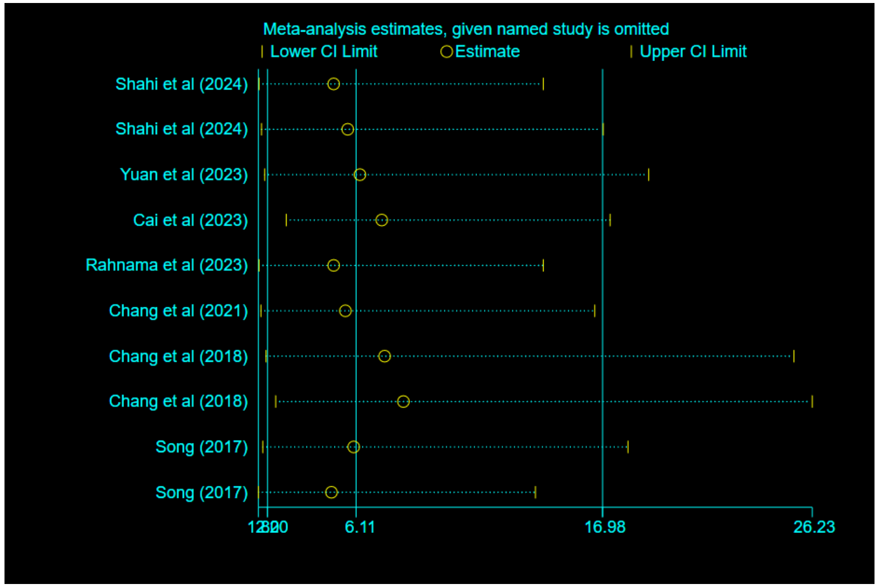

Supp Fig. 7. Sensitivity Analysis of MSC-sEVs on survival rate

Publication Bias

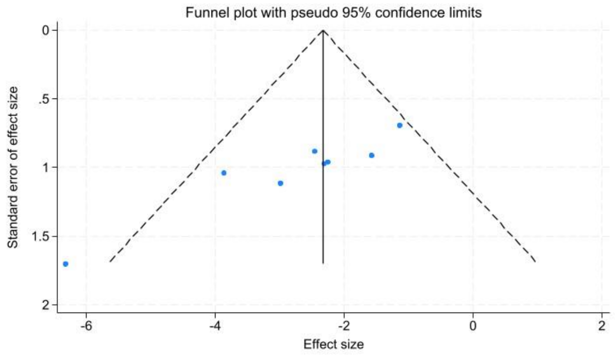

Supp. Fig. 8. Funnel plots for ALT

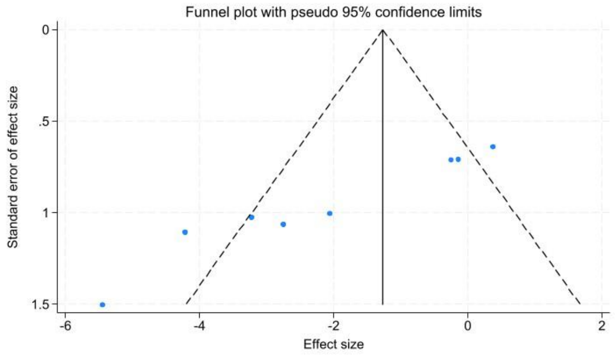

Supp. Fig. 9. Funnel plots for AST

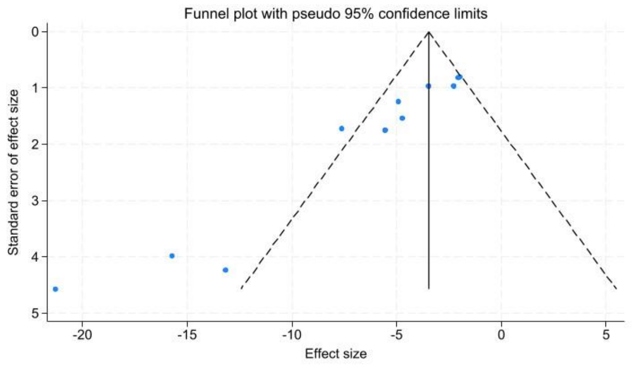

Supp. Fig. 10. Funnel plots for TNF-α

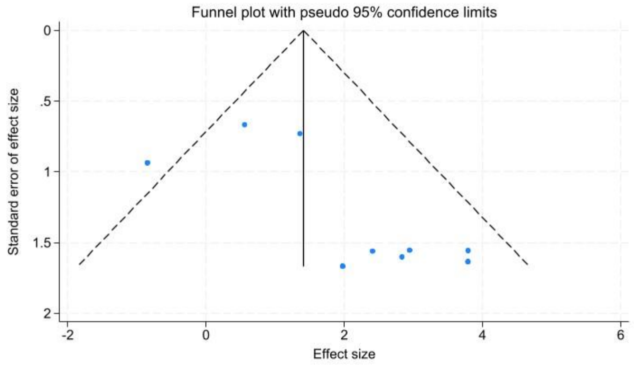

Supp. Fig. 11. Funnel plots for survival rate
